# Supplementary material for: RpNGS: an automated platform for pathogen identification and monitoring in clinical metagenomics data
Source: PeerJ. 2025 Aug 12;13:e19849. doi: 10.7717/peerj.19849 (PMC12356182; doi:10.7717/peerj.19849)
Supplement: Supplemental Information 1 [file peerj-13-19849-s001.pdf]

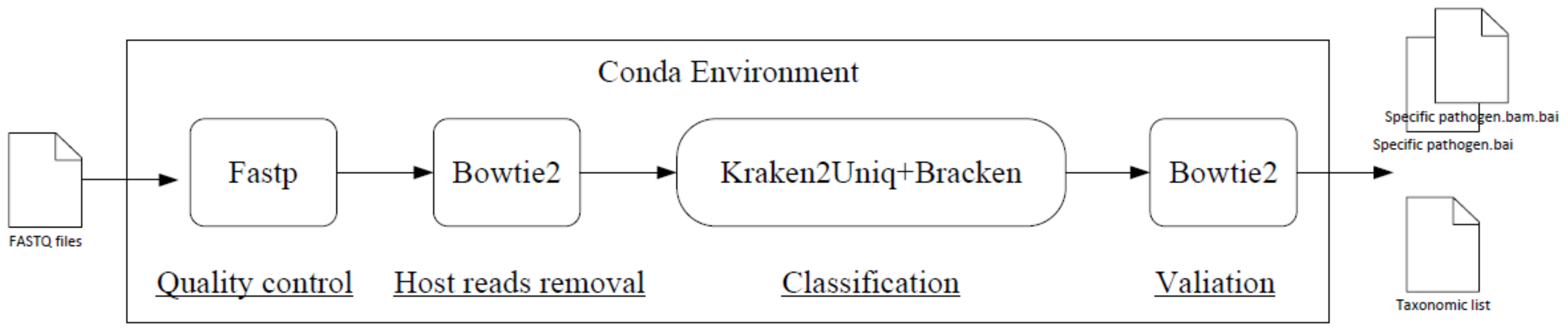

Fig S1 The analysis workflow for pathogen detection based on sequencing data.

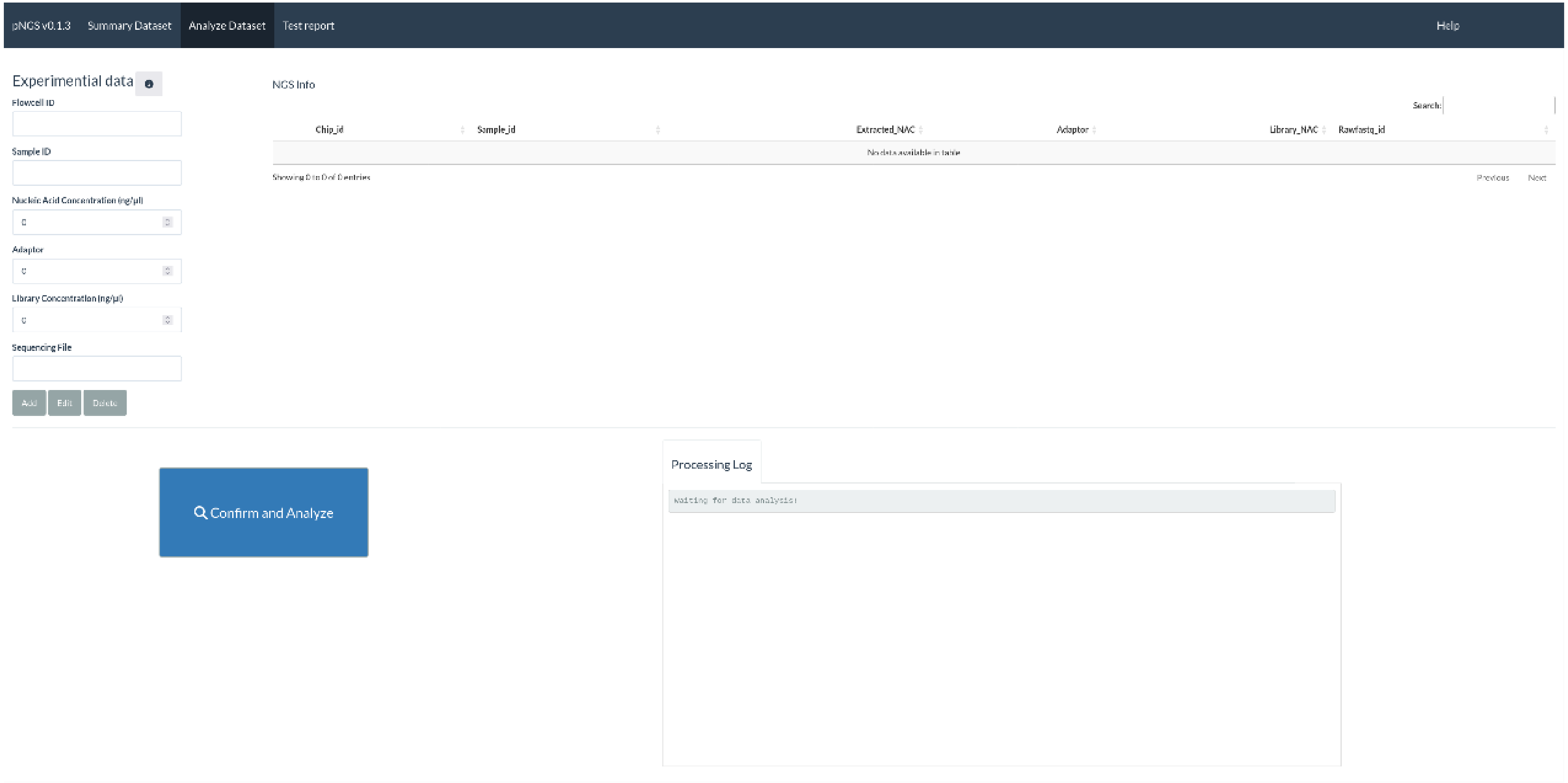

Fig S2 Updating the information of each batch including flow cell id, sample id, nucleic acids concentration after extraction and library preparation steps, adaptor id, and file name of sequencing data. Then start the process step by click the process button.

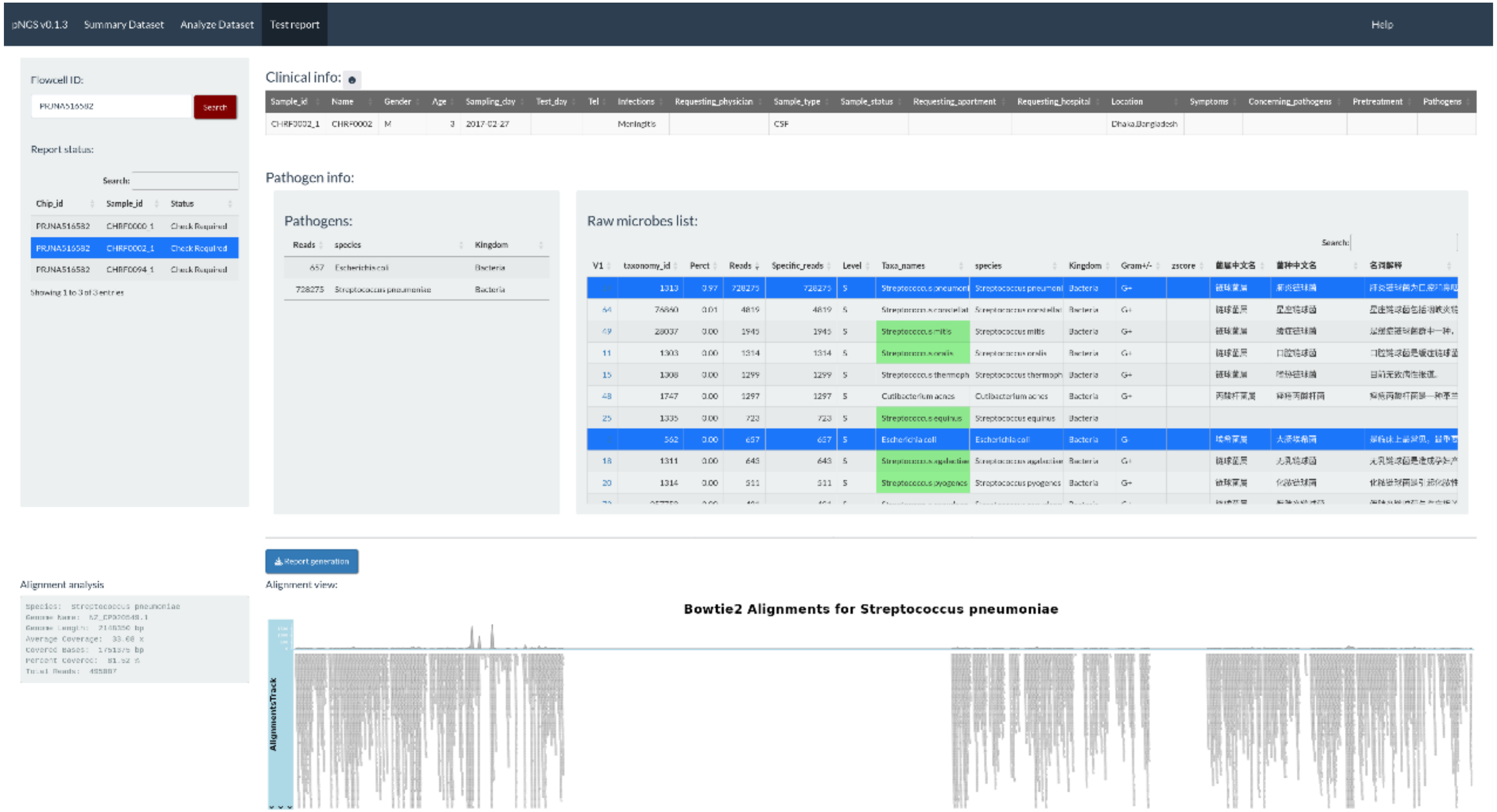

Fig S3 clinician or certified user, manually decide the pathogen for specific patient from microbes list based on multiple factors such as z-scores, mapped reads, average coverage for a specific pathogen, gender, age, sample types, clinical concerned pathogens types, anti-infection treatment. an mNGS detection report was generated with one click.

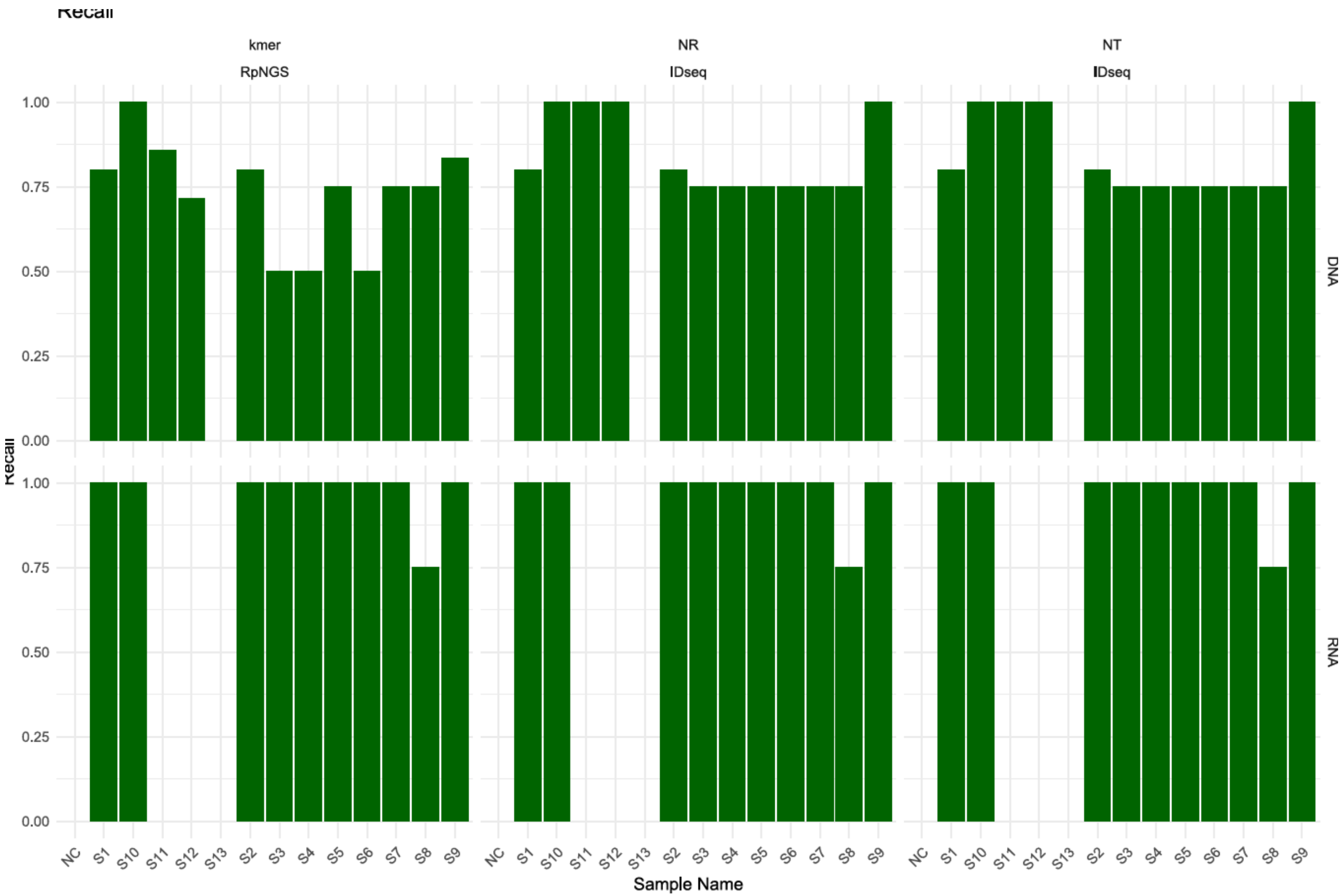

Fig S4 The Recall of IDseq_nt, IDseq_nr and RpNGS for DNA and RNA sequencing datasets among 14 samples.

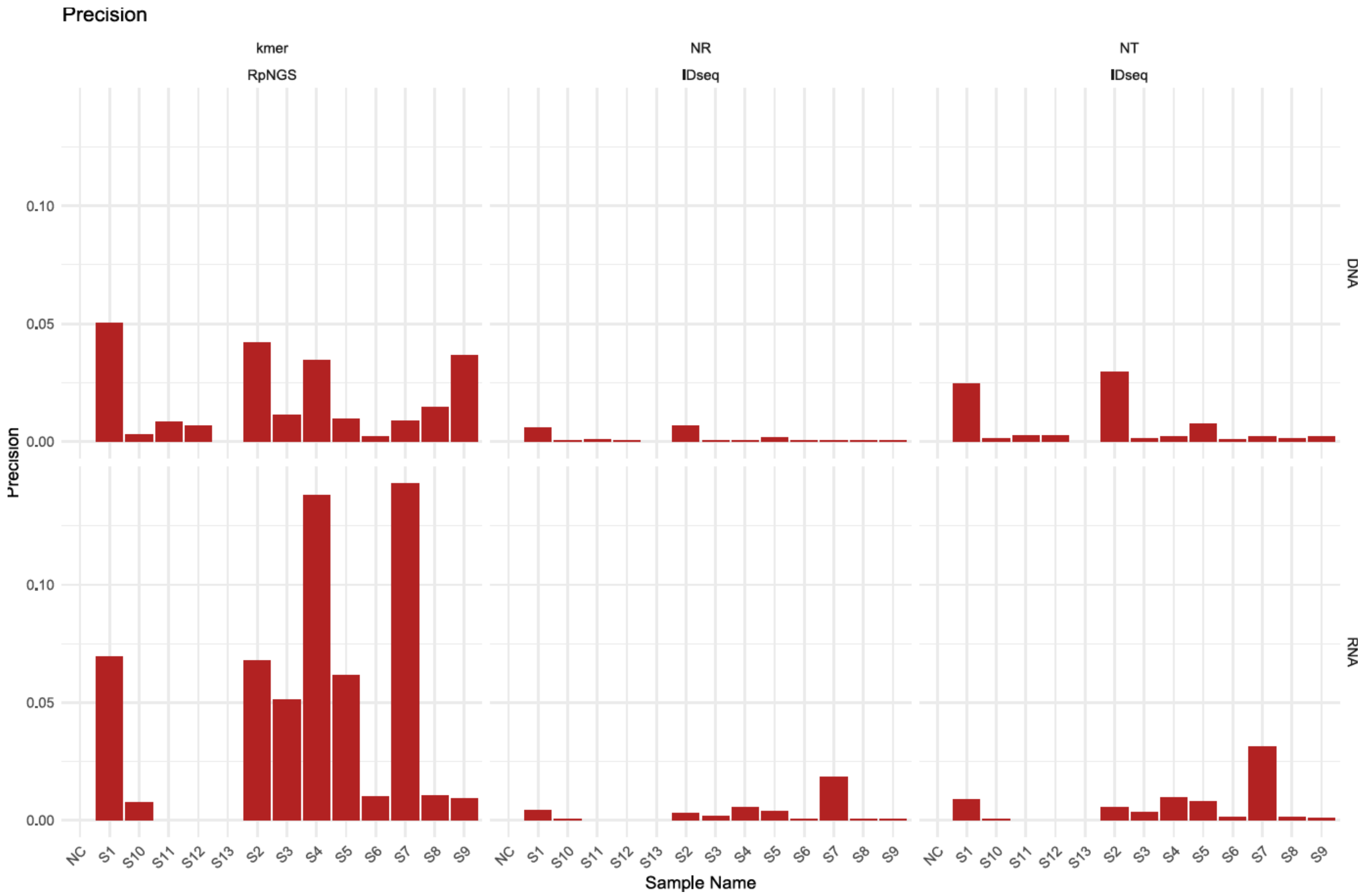

Fig S5 The precision of IDseq_nt, IDseq_nr and RpNGS for DNA and RNA sequencing datasets among 14 samples with z-score correction.

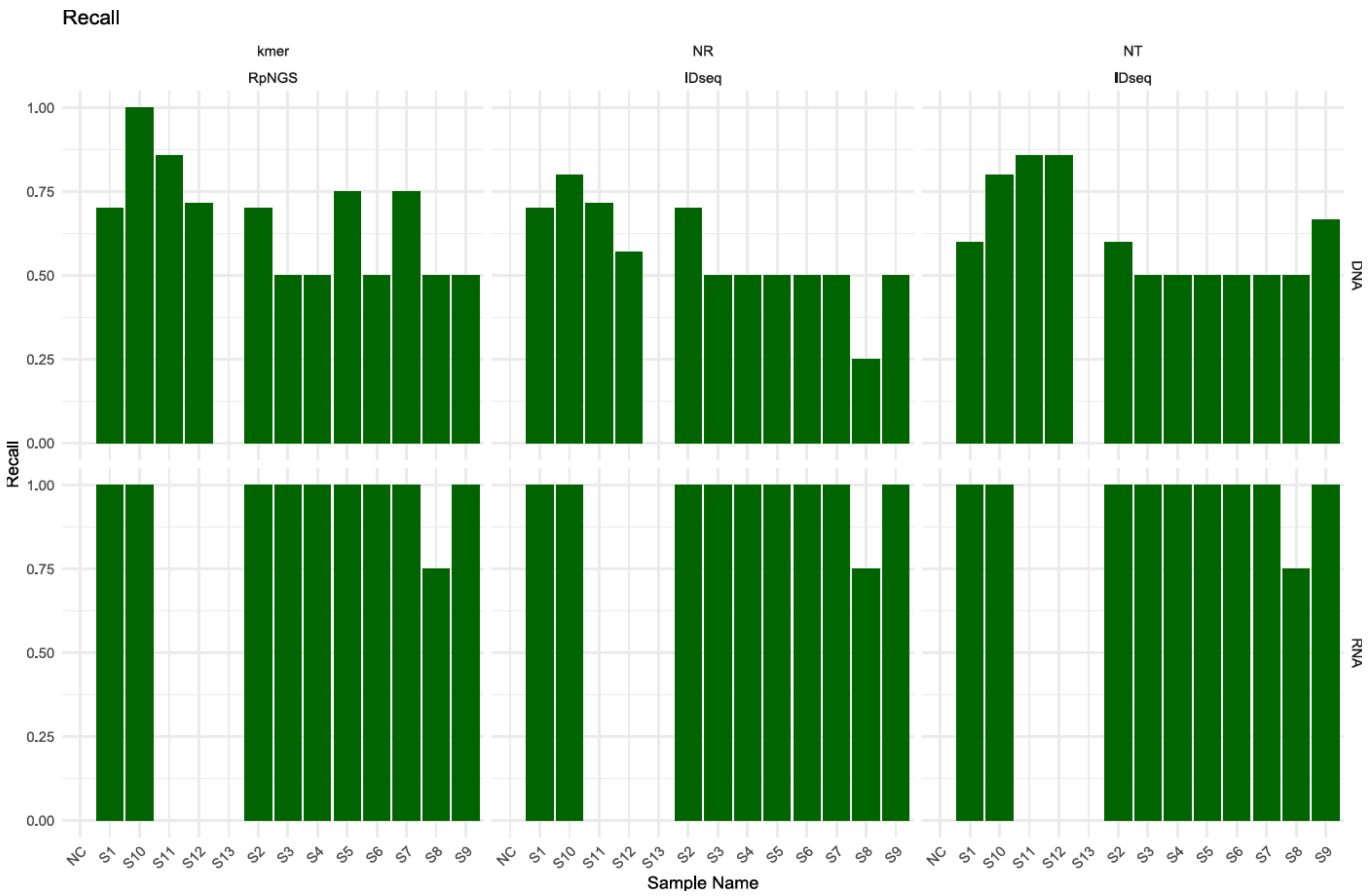

Fig S6 The Recall of IDseq_nt, IDseq_nr and RpNGS for DNA and RNA sequencing datasets among 14 samples with z-score correction.

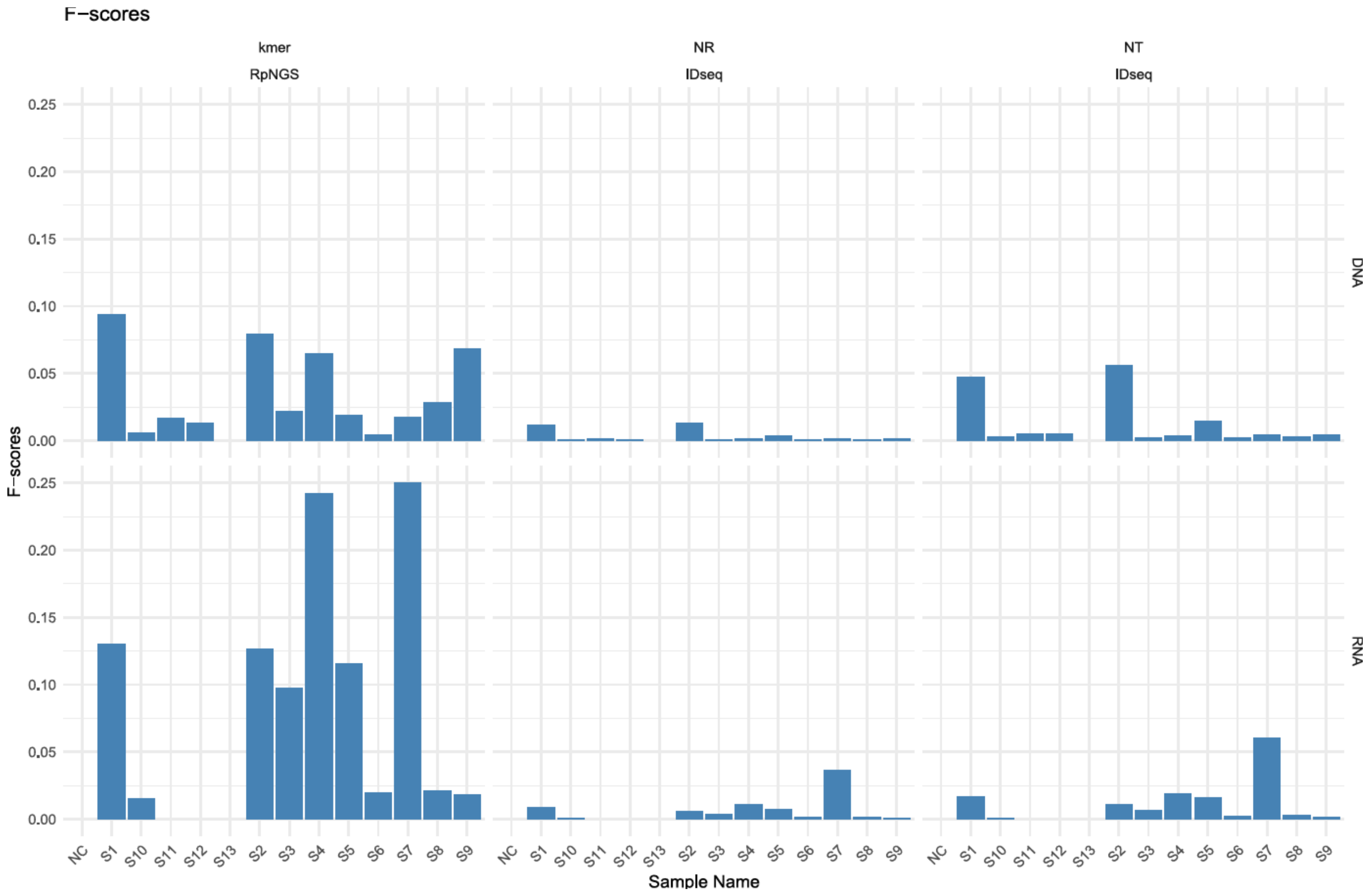

Fig S7 The F scores of IDseq_nt, IDseq_nr and RpNGS for DNA and RNA sequencing datasets among 14 samples with z-score correction.

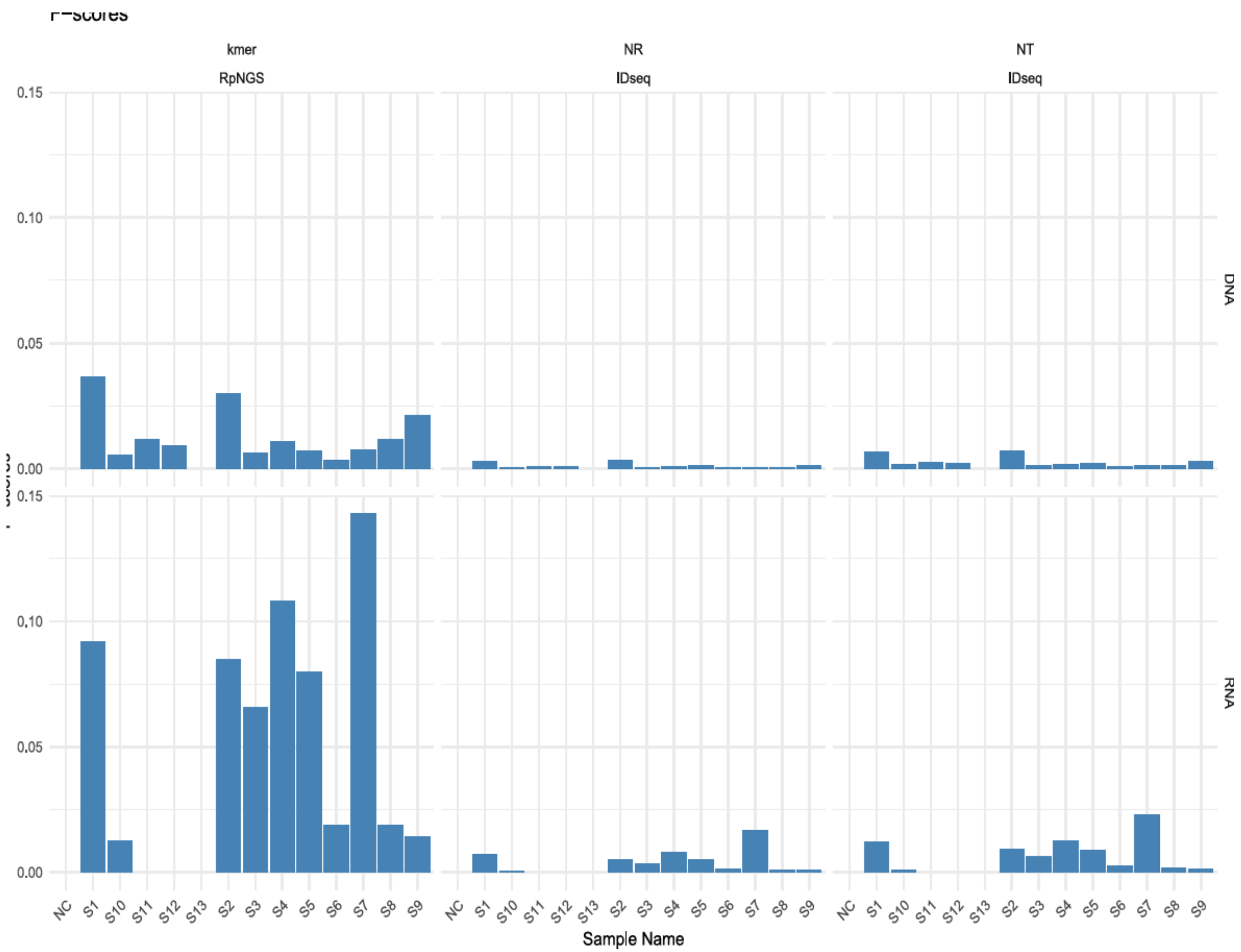

Fig S8 The F scores of IDseq_nt, IDseq_nr and RpNGS for DNA and RNA sequencing datasets among 14 samples without z-score correction.

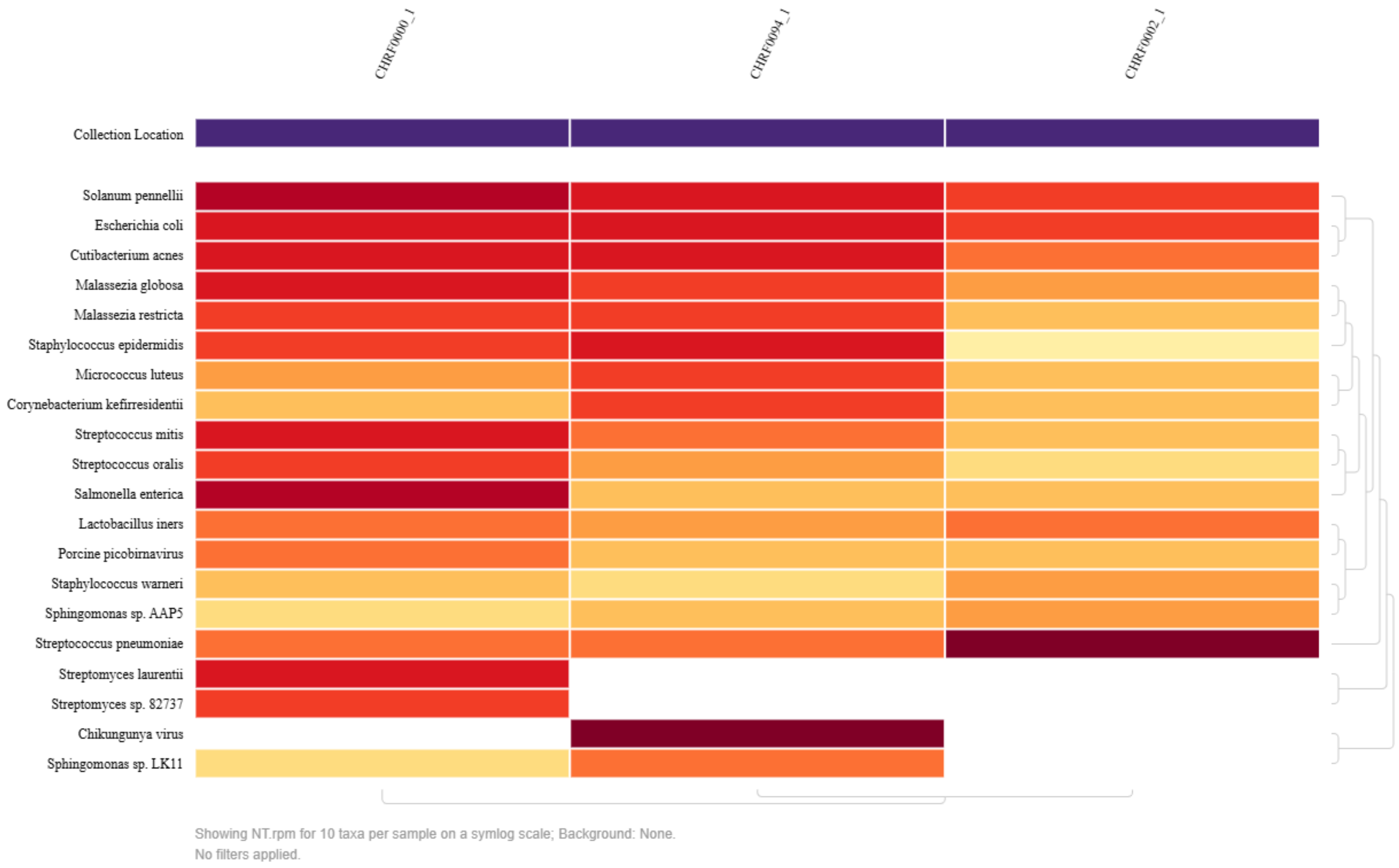

Fig. S9 Heatmap of species detected among these samples

Table S1 Basic characteristics of the microbes within the reference panel.

Microbes	Type	Genome Size (Mb)	GC%	Detailed information	S1	S2	S3	S4	S5	S6	S7	S8	S9	S10	S11	S12	S13	NC
Bacteroides fragilis	Gram-bacteria	5.22248	43.4	ATCC 25285	1.0×10^{2}	1.0×10^{2}	\	\	\	\	\	\	\	1.0×10^{2}	1.0×10^{2}	1.0×10^{2}	\	\
Betacoronavirus 1	RNA virus	0.03088	37	Clinical strain	1.0×10^{6}	1.0×10^{6}	\	\	\	\	\	\	\	\	\	\	\	\
Candida albicans	Fungus	14.6999	33.6	ATCC 10231	2.0×10^{4}	2.0×10^{4}	1.0×10^{2}	1.0×10^{3}	1.0×10^{4}	1.0×10^{3}	1.0×10^{3}	1.0×10^{3}	\	\	\	\	\	\
Cryptococcus neoformans	Fungus	18.5736	48.2	ATCC 66031	\	\	\	\	\	\	\	\	\	\	\	2.0×10^{4}	\	\
Enterococcus faecalis	Gram+ bacteria	2.96381	37.4	Clinical strain	5.0×10^{3}	5.0×10^{3}	\	\	\	\	\	\	\	\	\	\	\	\
Escherichia coli	Gram-bacteria	5.11174	50.6	ATCC 25922	\	\	\	\	\	\	\	\	1.0×10^{2}	\	1.0×10^{3}	1.0×10^{2}	\	\
Haemophilus influenzae	Gram-bacteria	1.8477	38	ATCC 49247	1.0×10^{5}	1.0×10^{5}	\	\	\	\	\	\	\	\	1.0×10^{2}	1.0×10^{4}	\	\

Haemophilus parainfluenzae	Gram-bacteria	1.96856	39.5	Clinical strain	1.0×10^{3}	1.0×10^{3}	\	\	\	\	\	\	1.0×10^{3}	\	\	\	\	\
Human coronavirus 229E	RNA virus	0.02732	38.3	ATCC VR-740	1.0×10^{4}	1.0×10^{4}	1.0×10^{3}	1.0×10^{4}	1.0×10^{5}	1.0×10^{4}	1.0×10^{4}	1.0×10^{4}	\	\	\	\	\	\
Human mastadenovirus C	DNA virus	0.03592	55.2	Clinical strain	1.0×10^{4}	1.0×10^{4}	1.0×10^{3}	1.0×10^{4}	1.0×10^{5}	1.0×10^{4}	1.0×10^{4}	1.0×10^{4}	\	\	1.0×10^{5}	\	\	\
Human metapneumovirus	RNA virus	0.01334	36.3	Clinical strain	1.0×10^{5}	1.0×10^{5}	\	\	\	\	\	\	\	\	\	\	\	\
Human orthopneumovirus	RNA virus	0.01519	33.6	Clinical strain	\	\	1.0×10^{3}	1.0×10^{4}	1.0×10^{5}	1.0×10^{4}	1.0×10^{4}	1.0×10^{4}	\	1.0×10^{5}	\	\	\	\
Human respirovirus 3	RNA virus	0.01546	34.9	Clinical strain	5.0×10^{5}	5.0×10^{5}	1.0×10^{3}	1.0×10^{4}	1.0×10^{5}	1.0×10^{4}	1.0×10^{4}	1.0×10^{4}	\	\	\	\	\	\
Influenza A virus	RNA virus	0.01313	43.6	Clinical strain	\	\	1.0×10^{3}	1.0×10^{4}	1.0×10^{5}	1.0×10^{4}	1.0×10^{4}	1.0×10^{4}	5.0×10^{5}	\	\	\	\	\
Klebsiella pneumoniae	Gram-bacteria	5.59628	57.2	ATCC BAA-1075	\	\	1.0×10^{2}	1.0×10^{3}	1.0×10^{4}	1.0×10^{3}	1.0×10^{3}	1.0×10^{3}	\	\	\	\	\	\
Primate bocaparvovirus 1	DNA virus	0.00542	42.3	Clinical strain	5.0×10^{4}	5.0×10^{4}	\	\	\	\	\	\	\	\	\	\	\	\

Pseudomonas aeruginosa	Gram-bacteria	6.63399	66.2	ATCC 27853	\	\	\	\	\	\	\	\	1.0×10^{5}	1.0×10^{3}	\	\	\	\
Rhinovirus B	RNA virus	0.00722	39.6	Clinical strain	5.0×10^{4}	5.0×10^{4}	\	\	\	\	\	\		\	\	\	\	\
Serratia marcescens	Gram-bacteria	5.22621	59.7	Clinical strain	\	\	\	\	\	\	\	\	1.0×10^{3}	1.0×10^{4}	\	\	\	\
Staphylococcus aureus	Gram+bacteria	2.82782	32.7	ATCC 43300	5.0×10^{4}	5.0×10^{4}	\	\	\	\	\	\	1.0×10^{2}	1.0×10^{3}	1.0×10^{3}	\	\	\
Staphylococcus epidermidis	Gram+bacteria	2.50584	32	Clinical strain	1.0×10^{3}	1.0×10^{3}	\	\	\	\	\	\	1.0×10^{3}	1.0×10^{4}	1.0×10^{2}	1.0×10^{3}	\	\
Stenotrophomonas maltophilia	Gram-bacteria	4.64215	66.4	Clinical strain	1.0×10^{2}	1.0×10^{2}	\	\	\	\	\	\	\	\	1.0×10^{3}	1.0×10^{4}	\	\
Streptococcus pneumoniae	Gram+bacteria	2.08598	39.6	Clinical strain	\	\	1.0×10^{2}	1.0×10^{3}	1.0×10^{4}	1.0×10^{3}	1.0×10^{3}	1.0×10^{3}	\	\	\	1.0×10^{3}	\	\
GM12878 cells	Host				2.0×10^{5}	2.0×10^{5}	2.0×10^{5}	2.0×10^{5}	2.0×10^{5}	2.0×10^{4}	2.0×10^{6}	\	2.0×10^{5}	2.0×10^{5}	2.0×10^{5}	2.0×10^{5}	2.0×10^{5}	2.0×10^{5}

Negative BALF samples	Host				\	\	\	\	\	\	\	2.0× 10^6	\	\	\	\	\	\

Table S2 Fastq files of S1-S13 and 1 negative control generated by lab005.

Accession	Run title	Experiment accession	Experiment title	Sample Name	BioSample accession	Platform	Read length
HRR1209919	Lab005_NC_DNA	HRX955813	Lab005_NC_DNA.fq.gz	NC	HRS775964	Illumina Nextseq 550	75bp
HRR1209920	Lab005_NC_RNA	HRX955814	Lab005_NC_RNA.fq.gz	NC	HRS775964	Illumina Nextseq 550	75bp
HRR1209921	Lab005_S1_DNA	HRX955815	Lab005_S1_DNA.fq.gz	S1	HRS775951	Illumina Nextseq 550	75bp
HRR1209922	Lab005_S1_RNA	HRX955816	Lab005_S1_RNA.fq.gz	S1	HRS775951	Illumina Nextseq 550	75bp
HRR1209923	Lab005_S10_DNA	HRX955817	Lab005_S10_DNA.fq.gz	S10	HRS775960	Illumina Nextseq 550	75bp
HRR1209924	Lab005_S10_RNA	HRX955818	Lab005_S10_RNA.fq.gz	S10	HRS775960	Illumina Nextseq 550	75bp
HRR1209925	Lab005_S11_DNA	HRX955819	Lab005_S11_DNA.fq.gz	S11	HRS775961	Illumina Nextseq 550	75bp
HRR1209926	Lab005_S11_RNA	HRX955820	Lab005_S11_RNA.fq.gz	S11	HRS775961	Illumina Nextseq 550	75bp
HRR1209927	Lab005_S12_DNA	HRX955821	Lab005_S12_DNA.fq.gz	S12	HRS775962	Illumina Nextseq 550	75bp
HRR1209928	Lab005_S12_RNA	HRX955822	Lab005_S12_RNA.fq.gz	S12	HRS775962	Illumina Nextseq 550	75bp
HRR1209929	Lab005_S13_DNA	HRX955823	Lab005_S13_DNA.fq.gz	S13	HRS775963	Illumina Nextseq 550	75bp
HRR1209930	Lab005_S13_RNA	HRX955824	Lab005_S13_RNA.fq.gz	S13	HRS775963	Illumina Nextseq 550	75bp
HRR1209931	Lab005_S2_DNA	HRX955825	Lab005_S2_DNA.fq.gz	S2	HRS775952	Illumina Nextseq 550	75bp
HRR1209932	Lab005_S2_RNA	HRX955826	Lab005_S2_RNA.fq.gz	S2	HRS775952	Illumina Nextseq 550	75bp
HRR1209933	Lab005_S3_DNA	HRX955827	Lab005_S3_DNA.fq.gz	S3	HRS775953	Illumina Nextseq 550	75bp
HRR1209934	Lab005_S3_RNA	HRX955828	Lab005_S3_RNA.fq.gz	S3	HRS775953	Illumina Nextseq 550	75bp
HRR1209935	Lab005_S4_DNA	HRX955829	Lab005_S4_DNA.fq.gz	S4	HRS775954	Illumina Nextseq 550	75bp
HRR1209936	Lab005_S4_RNA	HRX955830	Lab005_S4_RNA.fq.gz	S4	HRS775954	Illumina Nextseq 550	75bp
HRR1209937	Lab005_S5_DNA	HRX955831	Lab005_S5_DNA.fq.gz	S5	HRS775955	Illumina Nextseq 550	75bp
HRR1209938	Lab005_S5_RNA	HRX955832	Lab005_S5_RNA.fq.gz	S5	HRS775955	Illumina Nextseq 550	75bp
HRR1209939	Lab005_S6_DNA	HRX955833	Lab005_S6_DNA.fq.gz	S6	HRS775956	Illumina Nextseq 550	75bp

HRR1209940	Lab005_S6_RNA	HRX955834	Lab005_S6_RNA.fq.gz	S6	HRS775956	Illumina Nextseq 550	75bp
HRR1209941	Lab005_S7_DNA	HRX955835	Lab005_S7_DNA.fq.gz	S7	HRS775957	Illumina Nextseq 550	75bp
HRR1209942	Lab005_S7_RNA	HRX955836	Lab005_S7_RNA.fq.gz	S7	HRS775957	Illumina Nextseq 550	75bp
HRR1209943	Lab005_S8_DNA	HRX955837	Lab005_S8_DNA.fq.gz	S8	HRS775958	Illumina Nextseq 550	75bp
HRR1209944	Lab005_S8_RNA	HRX955838	Lab005_S8_RNA.fq.gz	S8	HRS775958	Illumina Nextseq 550	75bp
HRR1209945	Lab005_S9_DNA	HRX955839	Lab005_S9_DNA.fq.gz	S9	HRS775959	Illumina Nextseq 550	75bp
HRR1209946	Lab005_S9_RNA	HRX955840	Lab005_S9_RNA.fq.gz	S9	HRS775959	Illumina Nextseq 550	75bp

Table S3 RpNGS did not detect certain pathogens

	Undetected pathogens	kraken2uniq(reads)	kraken2uniq+bracken(reads)	IDseq_nt(reads)	IDseq_nr(reads)
S11	*Haemophilus influenzae*	6	NA	6	14
S12	*Haemophilus influenzae*	8	NA	13	35
S12	*Streptococcus pneumoniae*	4	NA	222	376
S3	*Streptococcus pneumoniae*	1	NA	2	137
S3	*Human mastadenovirus C*	NA	NA	NA	NA
S4	*Streptococcus pneumoniae*	2	NA	2	70
S4	*Human mastadenovirus C*	NA	NA	NA	NA
S6	*Streptococcus pneumoniae*	9	NA	17	441
S6	*Human mastadenovirus C*	NA	NA	NA	NA
S9	*Haemophilus parainfluenzae*	2	NA	3	3

Table S4 species detected among these samples

	CHRF0000	CHRF0002	CHRF0094
Chikungunya virus	0	0	50506
Corynebacterium kefirresidentii	0	0	119
Cutibacterium acnes	3332	1297	13227
Escherichia coli	11161	657	3304
Lactobacillus iners	197	118	136
Malassezia restricta	1512	160	3749
Micrococcus luteus	248	0	717
Salmonella enterica	2124	183	1826
Sphingomonas sp. AAP5	0	117	0
Staphylococcus epidermidis	140	21	617
Streptococcus mitis	62	1945	155
Streptococcus oralis	18	1314	185
Streptococcus pneumoniae	882	728275	0
